# Integrative Analysis of Rhythmicity: From Biology to Urban Environments and Sustainability

**DOI:** 10.3390/ijerph20010764

**Published:** 2022-12-31

**Authors:** Miha Moškon, Tadeja Režen, Matevž Juvančič, Špela Verovšek

**Affiliations:** 1 Faculty of Computer and Information Science, University of Ljubljana, 1000 Ljubljana, Slovenia; 2 Centre for Functional Genomics and Bio-Chips, Institute for Biochemistry and Molecular Genetics, Faculty of Medicine, University of Ljubljana, 1000 Ljubljana, Slovenia; 3 Faculty of Architecture, University of Ljubljana, 1000 Ljubljana, Slovenia

**Keywords:** rhythmicity analysis, rhythms, integrative analyses, transferability, computational framework, sustainability, circadian clock, urban rhythms

## Abstract

From biological to socio-technical systems, rhythmic processes are pervasive in our environment. However, methods for their comprehensive analysis are prevalent only in specific fields that limit the transfer of knowledge across scientific disciplines. This hinders interdisciplinary research and integrative analyses of rhythms across different domains and datasets. In this paper, we review recent developments in cross-disciplinary rhythmicity research, with a focus on the importance of rhythmic analyses in urban planning and biomedical research. Furthermore, we describe the current state of the art of (integrative) computational methods for the investigation of rhythmic data. Finally, we discuss the further potential and propose necessary future developments for cross-disciplinary rhythmicity analysis to foster integration of heterogeneous datasets across different domains, as well as guide data-driven decision making beyond the boundaries of traditional intradisciplinary research, especially in the context of sustainable and healthy cities.

## 1. Introduction

One of the oldest recorded interplays between rhythmic natural cycles and society was the annual flooding of the Nile River. The cyclical recurrence had a deep impact on society and culture as a whole, where the daily lives of individuals and the society, beliefs, religion, food production, measuring of time and political stability became dependent on—and interwoven with—rhythmic, and in this particular case, natural phenomena. The occasional disturbances in rhythm had a deep and profound effect on the existential aspects of everyday life (health and well-being, famine, political instability, etc.) and on spiritual life (individual stress and collective anxiety and changes in religious rituals and beliefs) in all classes in Egyptian society. However, the process has not only been a one-sided reaction to a natural phenomenon, but it has also resulted in spatial interventions that began to manage and impact the natural cycle. Egyptian civilisation responded to and harnessed the power and usefulness of the floods by managing the river and floods, building irrigation infrastructure, adapting building processes (availability of transport deeper inland) and site selection (closer to the river) to better correspond with the floods and their benefits and finally managing to completely change the influence the rhythm had on the way of life in the region by building the Aswan High Dam [1]. As Egyptians learned to live with and manage the Nile and its flooding, they also started to study the cycle, its intensity (e.g., with nilometers) and its immediate influence on yield and life. Studies of rhythmicity, disturbances and wide-reaching effects on the social structure, stability, habits and culture came later [2].

Rhythmic processes (i.e., processes that reflect periodic response) are pervasive in our environment. For example, circadian clocks display daily oscillations and regulate up to half of all genes in an organism [3,4]. Furthermore, their disruption could have several health implications, such as diabetes, immune deficiencies and cardiovascular disease [5,6], or it could present a sign of infection (e.g., see [7]). Therefore, investigating circadian rhythms and their disturbance is vital, as it improves our understanding of disease occurrence, progression and prevention. For example, monitoring of a body’s circadian rhythm could predict the development of metabolic or neurodegenerative disorders [8,9]. In recent years, much research has been devoted to the analysis of circadian rhythms, especially in the field of biology and medicine [10]. Detection and analysis of rhythmic patterns have also become an important aspect in other fields of research, such as healthcare, economy, urbanism, sustainability of the living environment and managing the problems that arise due to the ageing of the population. For example, circadian patterns of older adults’ telephone call records have been observed to be an indicator of geriatric depression [11]. Analysis of the number of labour onsets per specific hour in a day can guide management in the planning of midwifery and medical staffing [12]. Analysis of packet-level rhythms in the context of a computer network’s traffic is vital for efficient network management and quality of service (QoS) assurance [13]. Analysis of daily traffic patterns, their rhythmicity and their changes over time can reveal modifications of the population’s behaviour and their implications, even in the time of the global pandemic [14]. Rhythmicity analyses allow us to accurately assess the rhythmic trends of key performance indicators (KPIs) within urban environments and monitor and predict the consequences of specific interventions [15,16]. Moreover, today more than ever, heterogeneous types of (rhythmic) data are being acquired. Data are increasingly being collected by citizens or end users, applying various citizen science initiatives as well as smart and wearable sensor devices aimed at improving the quality of life or different biomedical applications (see, e.g., [17,18]). Therefore, the importance of rhythmicity analyses is increasing due to the large availability of data reflecting the rhythmic response, which can open and answer new research questions in fields such as healthy environments, well-being and disease prevention, management and treatment.

Rhythmic patterns arising in different domains might have different underlying mechanisms. However, biological rhythms, especially circadian rhythms, are mostly guided by the light changes between night and day. Furthermore, urban environments might disrupt these rhythms, and in the latter case, mechanisms guiding urban rhythms are not necessarily shared with the basic biological rhythms but still affect them.

Rhythmicity analysis is thus vital in different research fields. The goals of such studies are consistent among these fields, namely identifying rhythmic trends, assessing their parameters (e.g., period, amplitude and acrophase of oscillations and number of peaks per period) and comparing these trends among different conditions (i.e., comparative rhythmicity analysis). In this context, the goal is to increase the understanding of nature as well as artificial systems. Moreover, an exact analysis of the rhythmic response and its characterisation allows for the prediction of future events and their trends. Since datasets acquired in the context of different research fields might have significant disparities and exhibit distinct properties, diverse methods for the analysis of rhythmic data have been introduced in different fields. Several scientific fields still lag behind in such research, which presents an obstacle to their further progress. It is now time to address this problem with computational approaches that would aim to transfer the rhythmicity analysis methods from the fields where such methods are well established (but limited to a selected context) to an arbitrary scientific field. Moreover, a transferable approach for rhythmicity analysis would allow us to integrate the datasets obtained from different research disciplines using a comprehensive analytical framework to overcome traditional boundaries of domains and thus enhance the research potential of interdisciplinary cooperation.

**Figure 1:**
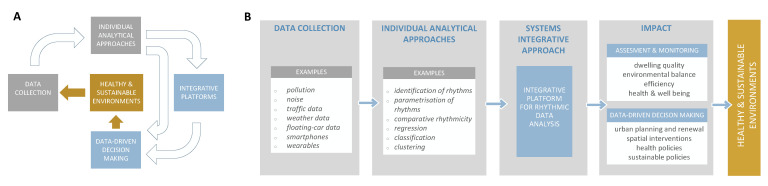
Computational framework for integrative analyses of rhythmic data. (**A**) The establishment of the proposed computational framework shall enable data-driven decision making guided beyond a single domain. (**B**) The application of the proposed computational framework would allow the implementation of comprehensive data-driven decision making in fostering sustainable and healthy urban environments.

In this paper, we present the gradual convergence and recognition of the analyses of rhythmic data as a tool for an in-depth insight into the rhythmic phenomena from the perspective of the two selected domains, namely biology and urbanism. We present the key similarities and the key discrepancies in the intensity of developments as tackled by the relevant literature on both sides, also evolving the discussion around the important advances in particular scopes of the given fields. Finally, we propose a computational framework devoted to integrative analyses of rhythmic data from different domains (see Figure 1). The proposed framework would allow us to identify correlations and causalities among factors governing environmental, urban and biological rhythms. Finally, such a framework would guide us in a systematic identification of the environmental factors responsible for the disruption of biological rhythms leading to disease occurrence and progression.

## 2. Analysis of Urban Rhythms

Rhythmicity as a theoretical and analytical concept in examining urban environments received attention decades ago after being introduced by several influential works in sociology, urban studies and geography (e.g., [19,20,21,22]). Drawing from within the sociological studies, Lefebvre [19] introduced and formulated rhythm as the interrelation between space, time and action or movement, whereas the urban setting is a polyrhythmic ensemble, congregating multiple overlapping rhythms of different scales of both natural and social origin [23]. Lefebvre makes references to everyday life and people’s routines in cities, which give hints and insights into where rhythm analysis could focus, pointing out examples of movement and traffic, exchanges of all kinds, sounds and colours, sudden events, festivity, rituals, the rhythm of moods, seasons, light and darkness, weather and urban functions or services [24]. Zerubavel [22] centred his research on examining social temporal regularities (i.e., the recurrent social patterns associated with social events and activities), pointing out the rhythms of daily life and reflecting on the use of urban spaces. Along with references to repetition and patterns, many authors pointed out the implication of change, transformation and irregular oscillations in rhythmic processes, increasing the need to detect and analyse how they differ in their recurrence and change over time [19,23,25]. In urban planning and architecture, the expressed question of rhythmicity is anchored in the modernist theorists who developed an interest in incorporating rhythm, repetition and cycles in spatial design [26] as a common approach for attaining design appeal and quality, as well as from an analytical perspective of the urban processes, such as the construction of city planning on the basis of the everyday shift between work time and spare time [27], the dichotomy between the individual and the collective, the rhythms of gathering and dispersal of the population [26] and the rhythm of the repeated urban elements and the consecutive visual sequences [28,29]. More recently, rhythmicity research has become pivotal in relation to the accelerated metropolisation, characterised by concentrated economic, political, cultural and environmental impact, bringing high densities in population, unprecedented entanglement of activities and intensity of use and movement [24,30]. Many of the phenomena and changes within such a complex environment, due to their repetitive nature, can be efficiently observed and tackled through rhythmic analyses. However, it was not until the last decade that the applicative urban analytics finally stopped struggling with constant data scarcity. Namely, the opportunities for research in the cities, whether of rhythmical or other trends, have rapidly increased due to new data-capturing technologies, mapping opportunities, sensors and the massive use of personal mobile devices in recent years.

The new knowledge of how to harness the power of massive data production, by mastering the way data are collected, organised, processed and analyzed, has brought shifts in decision making and the extension of the concept of rhythmicity by urban planners and policymakers, both from a methodological or procedural and practical perspective. Different questions on travelling and movement behaviour, accessibility evidence, trends of urban domestic or social activities, changes in socioeconomical structures, population and service distribution or the occurrences of new spatial use, the manifestation of (non)sustainable practices and the impact of recent policies are answered or observed nowadays through the rhythm analytics approach. However, the extensive and multidisciplinary field of city management and planning shows great differences in the progress of data-driven research within the domain. This is partly due to often weak integration and compatibility of data structures and their properties, as well as poorly exploited opportunities for transferring analytical methods, such as advanced rhythmic analysis, from other domains. Probably by far the most intensive application of rhythmicity in city-related analytics happened in examining the traffic flows, their predictability and time variance using reliable and coherent traffic data sources and their frequency. For instance, Jonathan et al. [31] applied hourly factors derived from traffic pattern groups that comprise count sites with similar hourly traffic variations and share features that generate these patterns. Rhythmic analysis shows how the classification of roads, traffic volume and land use characteristics helps explain hourly variations, which are important variables in the statistical clustering procedure and the factoring process. Wang et al. [32] developed a hybrid model of time variance, founded on filtering-based decomposition, and a least square support vector machine to attain accurate short-term traffic flow predictions in instances when it was hard to obtain a satisfactory result through the traditional methods (strong nonlinearity and non-stationarity in short-term traffic data). Furthermore, Yang et al. [33] extracted the characteristics of traffic variation in a larger metropolitan area by combining the rhythmicity analysis of the traffic speed dataset and the spectral clustering technique. In [16], the authors introduced a cosinor regression for the detection and characterisation of the rhythmicity of travel times based on short-term observations of travel times and traffic counts. Mon et al. [34] reported on the release of the extensive integrated dataset describing critical city inbound-outbound intersections, applying showcases of rhythmical analyses to support congestion estimation, traffic light control optimisation and the analysis of critical intersections. Similarly, in [35], the authors presented an open science initiative with extensive data collection captured by a swarm of drones aimed at transportation-oriented research and investigating traffic phenomena at different scales of rhythmic analyses and modelling. Kraft et al. [36] explored the daily mobility rhythms in an urban environment using data from intelligent transport systems. They focused on assessing the overall daily mobility and commuting rhythms in urban regions compared with the daily rhythms of individual locations within the examined area, with the goal of revealing the main conformities and differences.

Particularly interesting from the urban planning perspective are the studies that provide analysis based on integrated real-time or historic data sources such as floating car data or location data sourced by mobile operators’ datasets, coupled with other variables that reflect the human activity and end user attributes. Šveda et al. [37] explored the hourly mobile phone records of signalling exchanges by all major mobile network operators present in a certain urban area to monitor the spatiotemporal activities of the urban population and distinguish the typical rhythms of diurnal and nocturnal activity, resulting in the division of the urban area into relatively consistent territory types (chronopoles). Similarly, Su et al. [38] classified the streets according to the observed activity patterns based on the insights derived from high-resolution, anonymous and privacy-enhanced mobility data of street segments, and they revealed 10 distinct activity-based street types. Their examination of the activity rhythms showed that a street classification framework based on temporal street activity patterns can identify street categories at a finer granularity than other current methods, which can offer useful implications for state-of-the-art urban management and planning. Furthermore, in their study, Drevon et al. [39] addressed the rhythm-analytic approach to large urban events in order to confirm the impact of the event program and its structure on flows, oscillations between high- and low-intensity periods and the effects of the wider organisation of the leisure system. The circadian visualisation reveals the spread, which shows how the festival is integrated into the existing urban fabric, the openness, which shows accessibility, and the grip, which seeks to evaluate the wider influence of the event enriched by the addition of other data, including ticket scanning and commercial transactions.

The analysis of trends in the urban environment has become an important aspect of sustainability research and efficiency assessment (for example, see [40]). Rhythmicity assessment methods have only recently been directly integrated into sustainability analysis frameworks (for example, see [16]). It has been determined that various processes in our cities follow rhythmical patterns, and thus detailed analysis of rhythmicity (on a daily basis, weekly basis, seasonally, etc.) can extend the excavation of efficiency metrics, which depend on different factors (e.g., type of day (weekend or workday), weather conditions and time of year) and represent an important step in research supporting data-driven urban planning, responsive policies and management [37,39,41]. We should further extend these with the application of the proposed research in various monitoring scopes, from resource efficiency and sustainability to dwelling quality within smart city environments, with inputs ranging from vehicle count sensors, emissions and noise sensors, pedestrian sensing or passenger counts to other open repositories of governments or municipalities (e.g., energy consumption indicators or city overheating data or patterns) or citizen science-based infrastructures and their wider applicability. Therefore, analytical approaches and methods should support data-driven evaluation and decision making in urban planning and design and allow necessary deliberations on efficiency, resilience and public participation in planning practices. The proposed transparency of analytical methods, the results and the possible applications of the collected data from the citizens (e.g. Telraam’s WeCount traffic counting [42], Sensor. Community collective emissions sensing [43] and the UTD19 dataset [44]) will also promote responsible behaviour and more sustainable living patterns and opt to give residents and citizens an applicable role, expanding their scientific literacy. Wearable sensor devices that measure individual activity, well-being and health, as well as environmental parameters such as local air quality or noise perceptions, present another aspect of citizen-based data (see, e.g., [45]). These data have yet to be integrated into the cities’ KPI assessment frameworks. Furthermore, wearable sensor devices and the integration of the data they produce with the data describing the characteristics of the surrounding environment can be used to assess the correlations and causalities between environmental parameters and healthy living, such as those reflected in circadian patterns (e.g., sleep rhythms). We aim to address such integration of data obtained from different types of devices as well as different domains with the development of the proposed computational framework.

## 3. Analysis of Biological Rhythms in Health and Disease

The fundamental rhythms on Earth are governed by the rotation of the Earth along its axis, which defines the daily rhythms, and by the rotation of the Earth around the Sun, which determines the seasonal rhythms. The Earth’s cyclic rhythms influence all living organisms, which have evolved various biochemical, physiological and behavioural mechanisms to detect and adapt to cyclic changes in their environment. These biological rhythms enable organisms to adapt to cyclic changes in light, temperature, food resources, etc. One of the biological rhythms is the circadian rhythm, a rhythm of about one day (from the Latin *circa diem*) which is present in almost all living organisms, from photosynthetic bacteria to humans [46]. In mammals, the master circadian clock in the brain regulates the circadian rhythm of the body and its organs, or the peripheral circadian rhythm. The master clock is reset and synchronised to a 24 h light and dark cycle every day by the light entering the brain through the eyes [47]. The brain’s master clock regulates endocrine, immune and neuronal processes, resulting in rhythmic changes in body temperature, hormone secretion, sensitivity to infections or harmful chemicals, feeding and sleep behaviour, etc. [48].

Many environmental factors and human behaviour can affect the circadian clock, leading to disruption of the circadian rhythm and, in the long term, the development of various diseases. For example, the International Agency for Research on Cancer (IARC) has classified night-shift work that causes disruption of the physiological circadian rhythm as probably carcinogenic for humans [49]. The environmental factors that can cause disruption of the circadian rhythm also include environmental pollutants such as light and air pollutants that are present in urban environments. Light is the most potent synchronising factor for the brain’s master clock, and exposure to artificial light during the night leads to a disrupted circadian rhythm [50]. Numerous studies have found an association between artificial light at night and the incidence of various cancers, such as breast, colorectal and prostate cancer, in humans (recently reviewed in [50]). Outdoor air pollution also depends on traffic and its rhythm in urban environments. Exposure to elevated air pollution is associated with increased mortality from all causes, as well as higher incidence of respiratory and cardiovascular diseases and lung cancer [51]. The human body fights pollutants through different biochemical mechanisms controlled by the circadian rhythm. This means that the defence is lower or higher depending on the time of day. This also means that exposure to air pollutants at a more vulnerable time can lead to more damaging effects [52].

As there is growing evidence that disruption of circadian rhythms leads to lower performance and well-being in daily life and is associated with the development of disease, studies of circadian rhythms in humans are becoming increasingly important. Most studies of circadian rhythms have been performed on animal models and cell lines in a laboratory. Specialised equipment is used, and a variety of molecules can be measured at different time points every 24 h for several days, allowing precise analyses of rhythmicity. Transcriptome, proteome, acetylome and metabolome methods have been used to analyse several thousand molecules in time series experiments [53], and data along with the results of such analyses have been made available in public databases, such as CircaDB [54] and RhythmicDB [55]. The results of the analyses of experimental data, such as those reported in these resources, can be found using various computational methods specifically designed to identify and assess rhythmicity. In the context of the latter, we generally focus on the evaluation of the phase, amplitude, period and perturbations of circadian rhythms by different factors [56]. A variety of non-parametric (see, e.g., [57,58,59,60]) and parametric methods (see, e.g., [61,62,63,64]) have been proposed for circadian analysis of transcriptomics data. Each of these methods has certain advantages and pitfalls, and the selection of the most appropriate methods should be based on the characteristics of the analysed data set (e.g., sampling frequency, number of samples, sample balance), the shape of the rhythmic signal (e.g., single or multiple peaks per period) and the type of analysis (e.g., population-based or independent measurements). In addition, a robust assessment of rhythms is only possible if a sufficient number of samples is available for analysis. For example, the guidelines for circadian rhythm studies proposed by Hughes et al. recommend the acquisition of at least two complete cycles with a sampling interval of at least two hours [65].

The analysis of circadian rhythms in humans is challenging. First, it is too invasive to sample organs through biopsies at different time points over several days. We can only use minimally invasive methods, such as sampling body liquids (blood, saliva and urine) or measuring physiological processes with specialised medical devices. We can also analyse peripheral circadian rhythms in isolated human cells, such as peripheral blood mononuclear cells (PBMCs), hair and beard follicles and skin fibroblasts (recently reviewed in [66]). Analyses of the rhythmic concentration changes of various molecules in body liquids, such as melatonin, are performed in laboratories and require multiple samplings at different times of day for several days, including during the night [67]. Such experiments can be performed through separate visits by the subjects or by staying in a clinical laboratory, where we can control the light-dark and feeding rhythms and accurately measure heart rate and rhythm, sleep-wake rhythm, hormone and metabolite rhythms, etc. However, such experiments are expensive, time-consuming and impractical for studying circadian rhythms in larger cohorts.

One of the major breakthroughs in this field has been the development and widespread use of smartphones and wearables in the general population. These devices can record various types of rhythmic data such as the heart rate and rhythm, sleep-wake cycle and physical activity of millions of users. Using various mobile applications, we can measure and analyse the behavioural rhythms, feeding rhythms, menstrual cycles, etc. of millions of citizens. Users can monitor their physical fitness and health to improve their daily well-being. Due to their ease of use and wide applicability, wearables have quickly found their place in medicine as well. Remote medicine is a rapidly growing field that is harnessing the potential of commercial smart wearables to monitor patients remotely. Their use has already been tested for the screening and diagnosis of cardiovascular diseases [17] and kidney diseases [68] and for the monitoring of neuropsychiatric disorders, such as Parkinson’s disease [69]. However, there are still several challenges that need to be overcome, such as the limitations of sensor and computational technologies, approaches to data analyses, standardisation and privacy [17]. In a recent paper, it was shown that consumer-grade wearables can be used to detect changes in various rhythmic physiological processes, such as the circadian rhythm of the heart rate, to identify a healthy state from the onset of COVID-19 symptoms [70]. The importance of rhythm analyses in medicine is increasing because rhythmic data are widely available and can have a major impact on the design of a healthy environment and the prevention, management and treatment of diseases.

## 4. Integrative Analysis of Rhythmicity: From the Urban Environment to Biological Rhythms and Back

Even though several efforts have been made to identify and characterise rhythmicity in the data, these have been mostly limited to specific domains as well as to specific types of data. For example, a vast number of computational methods have been proposed in the context of identification and analysis of biological rhythms, especially in combination with gene expression (i.e., transcriptomics data (see a review of methods in [56,71])). However, these methods are not generally applicable or transferable to other kinds of data and express narrow applicability, which is not directly transferable to other scientific domains. Major discoveries have been fostered by interdisciplinary applications combing researchers with different backgrounds and working on different scientific domains. The current state of the art of rhythmic data analysis research does not allow the straightforward integration of rhythmic datasets from different domains, which would allow us to gain novel insights beyond the current knowledge.

An example of such integration could be found in the context of everyday patterns in the city. Relevant and available spatial and environmental (rhythmic) datasets can be linked with health-related (rhythmic) datasets, using transferable analytical approaches and seamless data integration. For example, vehicles’ daily traffic cycle along an urban arterial road is strongly affected by commuting patterns. These are linked to common working hours and service opening times in a particular area of a city. Traffic patterns are typically correlated with the air quality [72] and the noise intensity [73] in the residential areas along the road. Using the existent biomedical knowledge, we can hypothesise that rhythmic patterns of traffic along the road also affect and might disrupt the biological rhythms of residents. This hypothesis can be validated and further elaborated upon by correlating the rhythms describing daily traffic patterns with the biological rhythms of individuals along the road. However, such integration first requires a collection of data describing the environmental conditions (e.g., air quality, noise levels and weather conditions), traffic conditions (e.g., number of vehicles per hour) and physiological conditions of individuals (e.g., obtained using wearable devices). Moreover, it requires the integration of heterogeneous data sources, which are also generated and collected by the end users, to guide their comprehensive analyses for scientific, commercial or professional purposes in different domains.

Integrative analyses, as described above, require a set of methods that could be directly applicable to different scientific fields. Moreover, methods tend to be specific for a given type of data and have several presumptions (for example, presuming a single peak per period, data homoscedasticity or only working with either count or continuous data). The analysis of circadian transcriptomics datasets presents one of the most developed fields of rhythmic data research. In this context, several parametric methods, mostly based on a cosinor model, have been reported. Moškon described a comprehensive Python library with a suite of cosinor-based analyses suitable for continuous as well as count data analysis [62]. De los Santos et al. reported on an extension of the cosinor method, denoted as ECHO [63], as well as its integration into a methodology for multi-omics data analysis, denoted as MOSAIC [64]. Pelikan et al. [74] and Parson et al. [75] reporting a computational framework for comparative analysis of biological rhythms. Several non-parametric methods for the analysis of rhythmicity in transcriptomics data have also been reported. These include JTK Cycle [76], RAIN [58] and BooteJTK [57]. Recently, Ness-Cohn and Braun proposed a topology-inspired method for rhythmic transcript detection, denoted as TimeCycle [77]. A plethora of methods for detection and analysis of circadian patterns in transcriptomics data exists. However, each of these has several pitfalls, and each of them is especially well-suited for specific scenarios. This means that even when working in a domain with a wide availability of rhythmicity analysis methods, one must select a method that is tailored to work (or is optimal to work) with a specific type of input [78]. The properties of the analysed dataset should thus guide the selection of the most suitable method (e.g., the sampling frequency, the number of obtained samples, sample balance, (a)symmetry of a waveform, number of peaks per period and presence of replicates) and by the type of the analysis (e.g., population or independent measurement analysis) [65,79]. Moreover, the transparent integration of methods and available datasets shall enable the recognition of suitable metrics that allow for valid comparisons and benchmarking (e.g., in time or geo-wise) or the possible interchange of the dataset (or the indicator) in cases where record gaps occur. Finally, the key features of the integrative platform shall include a selection among the vast scope of methods which allows the identification of the method that yields the best results for a given dataset in a given context.

## 5. Conclusions

Integrative analyses are also becoming vital in the field of analysis of rhythmic data. This aspect is gaining relevance in the field of urbanism, where a sustainable and healthy environment needs to be analysed and planned as a whole, namely from a systems perspective [80]. However, we lack a set of computational tools that would allow us to perform straightforward integrative analyses of heterogeneous rhythmic datasets with the included capabilities of (1) comprehensive implementation of a wide scope of state-of-the-art rhythmicity detection methods applicable across different types of data, (2) automated (or at least guided) identification of the most suitable method for a dataset, (3) validation of the obtained results with regard to available knowledge, (4) integration of the results from different datasets to obtain correlations and possible causalities among different factors, and finally (5) ease of use, data importing and presentation of the results in an interpretable format. Some efforts have already been made in these directions. For example, Carlucci et al. proposed the computational framework called DiscoRhythm for performing rhythmic analyses of gene expression data on an omic scale with an easy-to-use web interface [81]. The framework supports the execution of five previously reported methods, namely cosinor [61], ARSER [82], JTK Cycle [76], Lomb-Scargle [83] and MetaCycle [84]. More recently, Brooks et al. proposed a similar framework denoted as NiteCap, incorporating additional rhythmicity analysis methods as well as different methods to compare differential rhythmicity across two conditions [85]. However, both of these approaches were focused only on the analysis of gene expression data. An approach towards a domain-agnostic framework devoted to the analysis of biological as well as non-biological datasets, denoted as rhythmic data analysis (RDA), was proposed by Vestu et al. [86]. Even though the latter presents a comprehensive approach towards integrative analysis of rhythms, it is missing functionalities, such as automated selection of the most suitable method per dataset, implementation of additional, more robust methods applicable across different types of data, implementation of additional data analysis pipelines (e.g., data preprocessing, clustering, regression and classification) to integrate these with rhythmicity analyses and availability of an easy-to-use user interface.

The above-described points clearly designate several possible future research directions. First, addressing better interoperability of data, limitations deriving from the diversity of data subjects call for a structured and methodical integration of multiple data sources, which can be interlinked and widely applicable across different domains, and geographical entities by adhering to transparent and open data principles, offering a set of generic and semi-automated strategies for solving case-specific problems. Second, future efforts for a deliberated generalisation of the selected analytical methods are important to allow their application across fragmented datasets. Moreover, this also dictates the development of technical functionalities and guidance in the selection of the most suitable method for a given dataset and a given context. Namely, the collection of transferable rhythmicity analysis methods and datasets will command, by their characteristics, further settings of functionalities (automated or semi-automated) as an aid for end users. Future applicative research shall also propose tools to benchmark the implemented methods on datasets from different domains as well as synthetic datasets mimicking the data from different domains. Additionally, wider availability of such tools under open-source policies shall enable the users to apply and adapt these according to their needs. Third, the important direction for future research is a systematic approach towards reusing and integrating machine-readable, citizen-provided data in the decision-making procedures in urban planning. Namely, one of the prerequisites for planning healthy cities is a clear insight into the state of the environment and citizens’ health and well-being. Both require the examination of socioenvironmental systems on a finer granularity level than those achieved by traditional procedures. Systematisation of new approaches is equally important while bringing possibilities for time-wise and space-wise comparisons, increasing the possibilities for coupling the official datasets with targeted citizens’ data methodically and reliably and introducing robust procedures for data verification and usability.

These functionalities should be implemented in the near future to allow the integration of results of analyses on different datasets from different scientific domains (see Figure 1). Among the functionalities of the platform, as discussed in this paper, we consider the transfer of the advanced rhythmical analyses from biology to the city-related phenomena one of the vital contributions that can be initiated by the proposed platform. Finally, this will open many possibilities to identifying correlations and causal relations among observed factors ranging across different scientific disciplines. For example, integrative analyses, as proposed here, will offer further insight into the dynamics of rhythmic patterns in the environment (such as circadian traffic trends, seasonal temperature and air pollution variations, noise-load patterns and artificial lighting alterations) and their associations with various physiological states and the disruption of biological clocks affecting citizens’ health. Such research will systematically improve the interpretative strength of data fragments and develop assessment methods to inform policies and practices and support data-driven decision making in fields including transport and mobility, energy consumption, quality of public places or exploring the nexus between place, health and well-being.

## Data Availability

Not applicable.

## Abbreviations

The following abbreviations are used in this manuscript:

IARC	International Agency for Research on Cancer
KPI	Key performance indicator
QoS	Quality of service
PBMC	Peripheral blood mononuclear cell
RDA	Rhythmic data analysis
